# Interface Engineering of Styrenic Polymer Grafted Porous Micro-Silicon/Polyaniline Composite for Enhanced Lithium Storage Anode Materials

**DOI:** 10.3390/polym16243544

**Published:** 2024-12-19

**Authors:** Yechan Lee, Mahesh Naikwade, Sang-Wha Lee

**Affiliations:** Department of Chemical and Biological Engineering, Gachon University, 1342 Seongnamdaero, Sujeong-Gu, Seongnam-Si 13120, Gyeonggi-do, Republic of Korea; yech314@gmail.com (Y.L.); maheshnaikwade77@gmail.com (M.N.)

**Keywords:** micro-sized Si, low-temperature thermolytic grafting, Li-ion battery

## Abstract

Si anode materials are promising candidates for next-generation Li-ion batteries (LIBs) because of their high capacities. However, expansion and low conductivity result in rapid performance degradation. Herein, we present a facile one-pot method for pyrolyzing polystyrene sulfonate (PSS) polymers at low temperatures (≤400 °C) to form a thin carbonaceous layer on the silicon surface. Specifically, micron silicon (mSi) was transformed into porous mSi (por-mSi) by a metal-assisted chemical etching method, and a phenyl-based thin film derived from the thermolysis of PSS formed a strong Si–C/Si–O–C covalent bonding with the Si surface, which helped maintain stable cycle performance by improving the interfacial properties of mSi. Additionally, PSS-grafted por-mSi (por-mSi@PSS) anode was coated with polyaniline (PANI) for endowing additional electrical conductivity. The por-mSi@PSS/PANI anode demonstrated a high reversible capacity of ~1500 mAh g^−1^ at 0.1 A g^−1^ after 100 cycles, outperforming or matching the performance reported in recent studies. A thin double layer composed of phenyl moieties and a conductive PANI coating improved the stability of Si-based anodes and provided an effective pathway for Li^+^ ion transport to the Si interface, suggesting that polymer-modified Si anodes hold significant promise for advanced LIB applications.

## 1. Introduction

Li-ion batteries (LIBs) have emerged as a leading energy storage solution owing to their long cycle life, high energy density, and environmental suitability, among other favorable characteristics [1,2,3,4]. However, it is difficult for conventional C-based anode materials to meet the needs of electric vehicles and energy storage systems because of their low energy density and limited output capacity [5,6,7]. Despite the potential of Li metal as an ideal anode material owing to its high specific capacity, dendrite formation during the charging and discharging process shortens the electrode interior and poses significant risks [8,9,10]. Therefore, innovative approaches such as new nanostructures or advanced surface modifications are urgently required to improve the performance of electrode materials and develop high-capacity and high-power LIBs.

Various anode materials have been reported to replace graphite. In particular, Si is economical because of its abundance on Earth; furthermore, it stands out as a promising anode material for next-generation LIBs owing to its theoretical capacity and attractive operating voltage (0.5 V vs. Li/Li^+^) characteristics, which are more than 10 times that of graphite anodes [11,12,13,14]. However, its practical use is hindered by fatal problems such as poor electrical conductivity of Si (<10^−3^ S cm^−1^ at 25 °C) and significant volume expansion of about 300% during charge–discharge cycles [11]. In particular, repeated volume changes cause mechanical stress, resulting in electrode crushing and peeling off from the current collector and the formation of unstable solid electrolyte interfaces (SEIs) [15,16]. To address these issues, various Si nanostructures have been introduced to relieve the stress associated with volume changes in Si [17,18,19]. However, despite these efforts, the development of high-power and high-capacity batteries based on Si anodes remains challenging.

Recently, various methods have been proposed to physically suppress volume expansion by carbon coating on the Si surface [20,21,22,23,24]. Graphene and MXene are promising carbon-based 2D materials that are applicable to Si anodes due to their compliant mechanical strength and excellent conductivity [25,26,27,28]. However, most of these methods require high synthesis temperatures, making them economically unfeasible. Furthermore, the Si composite with high C content exhibits a significantly lower capacity than a pure Si anode. Consequently, this method is unsuitable for realizing the advantages of Si while maintaining its economic feasibility.

In this paper, we report a simple strategy for the synthesis of porous silicon composites by pyrolysis grafting and conductive polymer coating for improved Li storage anode materials. Specifically, micro-sized bulk silicon (mSi, ca. 5 μm) was modified into porous mSi (por-mSi) through a metal-assisted chemical etching (MACE) method [29]. Thereafter, a polystyrene sulfonate (PSS) solution was infiltrated into the por-mSi and pyrolyzed at a temperature below 400 °C to form a phenyl-based thin film on the por-mSi (por-mSi@PSS). Subsequently, conductive polyaniline (PANI) was coated onto por-mSi@PSS to obtain a por-mSi@PSS/PANI composite. The resulting composite suppressed the volume expansion of Si by grafting abundant phenyl groups via the Si–C/Si–O–C covalent linkages [30,31]. Furthermore, a very thin layer with a thickness of 4–5 nm functioned effectively without significantly reducing the specific capacity of the Si composite [32]. The coated PANI layer enhanced the electrical conductivity of the Si anode and acted as an additional binder through electrostatic attraction [33,34]. As a result, por-mSi@PSS/PANI showed a good cycling performance, maintaining a capacity of 1820 mAh g^−1^ after 50 cycles at 0.1 A g^−1^, which is remarkably better than that of pristine mSi (140 mAh g^−1^), por-mSi (903 mAh g^−1^), and por-mSi@PSS (1483 mAh g^−1^). The por-mSi@PSS/PANI also demonstrated an impressive capacity of 1077 mAh g^−1^ at 4 A g^−1^ and a high recovery capacity of 89.5% when returned to 0.1 A g^−1^.

## 2. Materials and Methods

### 2.1. Chemical Materials

Micro-sized Si powder (purity 99.9%, ~5 μm) was obtained from Kojundo (Uiwang, Republic of Korea). Poly(sodium 4-styrenesulfonate) (Mw~70 kDa), high-surface-area-conducting PANI, and an HF solution (48%, 7664-39-3) were sourced from Sigma-Aldrich (Seoul, Republic of Korea). AgNO_3_ powder was purchased from Duksan Pure Chemicals (Ansan, Republic of Korea). HNO_3_ solution (70 wt%) and anhydrous ethanol (99.9% purity) was obtained from Daejung Chemicals and Metals Co., Ltd. (Siheung, Republic of Korea). All the chemicals were used as received without further purification.

### 2.2. Synthesis of por-mSi

The MACE method using AgNO_3_ as a silver precursor was employed to fabricate the por-mSi particles [35]. After dispersing 2 g of Si powder in 50 mL of deionized (DI) water, the etching solution consisting of 5 M HF and 0.06 M AgNO_3_ was added dropwise to induce the oxidation of the Si surface by Ag, followed by the dissolution by HF solution, and this process proceeded for 1 h. The Ag-deposited Si particles were filtered and rinsed several times with DI water. The residual Ag in the particles was dissolved in 200 mL of HNO_3_ (70%) under constant stirring for 2 h. The products were then filtered and rinsed with DI water and ethanol to obtain por-mSi.

### 2.3. Preparation of por-mSi@PSS and por-mSi@PSS/PANI

The por-mSi@PSS sample was synthesized via a facile one-pot low-temperature pyrolysis process [36]. To remove the inevitably generated SiO_x_ layer, the por-mSi samples were treated with an HF solution (5 wt% in ethanol) for 10 min before use and then dried in a vacuum oven. Then, after activation by heating at 70 °C for 5 min on a hot plate, 1 mL of PSS solution (5 wt% in water) was dropped and deposited evenly on por-mSi. All solvents were evaporated at approximately 100 °C and then purged with N_2_ for 30 min in a quartz tube furnace, followed by heating at 360 °C for 1 h. After the reaction was completed, the samples were dispersed in DI water for 2 h to remove unreacted polymers, rinsed with water and ethanol, and dried in a vacuum oven at 60 °C. The prepared por-mSi@PSS was treated with an HF solution before use to remove SiO_2_.

The por-mSi@PSS/PANI sample was prepared by a simple coating method. The por-mSi@PSS sample treated with the HF solution was stirred in a conductive PANI solution (0.3 wt% in water) for 24 h. The mixture was centrifuged and dried to obtain the final composite. Scheme S1 summarizes the synthesis strategy for the thermolytic grafting and conductive polymer coating of por-mSi@PSS/PANI.

### 2.4. Characterization

The surface morphology and elemental distribution of each sample were investigated using field-emission scanning electron microscopy (FE-SEM; Hitachi-SU8600, Tokyo, Japan) coupled with energy-dispersive X-ray spectroscopy (EDS). High-resolution transmission electron microscopy (HR-TEM, Tecnai by FEI, Field Electron and Ion Company, Hillsboro, OR, USA) was used to observe the interface of Si materials. The crystal structures of the samples were analyzed by X-ray diffraction (XRD, Rigaku, Tokyo, Japan) with Cu Kα radiation (λ = 1.54 Å) at 30 kV and 15 mA in the 2θ range of 10–90° at a scan rate of 4° min^−1^. X-ray photoelectron spectroscopy (XPS, K-alpha+, Thermo Fisher Scientific, Waltham, MA, USA) was carried out using an Al Kα (hν = 1486.6 eV) source and a pass energy of 15 kV. Raman spectroscopy (Monora 500i ANDOR, Oxford Instruments, Abingdon, UK) with 632 nm laser excitation was used to determine the Raman spectra of the samples. Thermogravimetric analysis (TGA, Q600, TA Instruments, New Castle, DE, USA) of the samples was performed under N_2_ flow at a heating rate of 10 °C min^−1^ from room temperature (RT) to 800 °C.

### 2.5. Electrochemical Measurements

The electrode was prepared by mixing 60 wt% of an active material (Denka Black 100, Denka, Seoul, Republic of Korea) with a 20 wt% polyacrylic acid solution (PAA, Sigma-Aldrich, Seoul, Republic of Korea, 10 wt% in ethanol). The uniformly mixed slurry was cast on Cu foil using a doctor blade. The electrode was then dried in a vacuum oven at 60 °C for 24 h. The mass loading of the active material was between 1.00 and 1.15 mg cm^−2^, with a measured thickness of 16 ± 0.5 μm. The electrode was then cut using a 1.2 cm diameter punch. Electrochemical measurements of the cathode active material were performed using a CR2032 coin-type half-cell. Li metal (Wellcos Co., Seoul, Republic of Korea) was used as the counter electrode, a microporous polypropylene (PP) membrane (Celgard 2400, Celgard, Charlotte, NC, USA) was used as the separator, and 1.0 M LiPF_6_ in ethylene carbonate:diethyl carbonate (5:5 *v*/*v*) containing 5 wt% fluoroethylene carbonate (FEC) was used as the electrolyte. The cells were assembled in a glove box purged with high-purity Ar gas. The electrochemical performance was evaluated within the potential range of 0.01–1.5 V (vs. Li/Li^+^) at various current densities. Cyclic voltammetry (CV) and electrochemical impedance spectroscopy (EIS) were performed using a ZIVE SP1 (Zivelab, Seoul, Republic of Korea) single-channel potentiometer. CV data were obtained with a voltage window of 0.0–1.5 V with a scan rate of 0.2 mV s^−1^, and an EIS analysis was performed with a frequency range of 10^5^ to 10^−3^ Hz with an amplitude perturbation signal of 5 mV using the Smart Manager software (ver. 6.748, Zivelab, Seoul, Republic of Korea) package. Nyquist plots for equivalent circuit modeling were fitted using Z-View software (ver. 4.0, Scribner Associates, Southern Pines, NC, USA). Rate capability tests were performed at current densities ranging from 0.1 to 4 A g^−1^ with each density maintained for five cycles. The capacities of all the coin cells were calculated based on the mass of Si present in the active material.

## 3. Results and Discussion

### 3.1. Preparation and Characterization of pSi Composites

**Figure 1:**
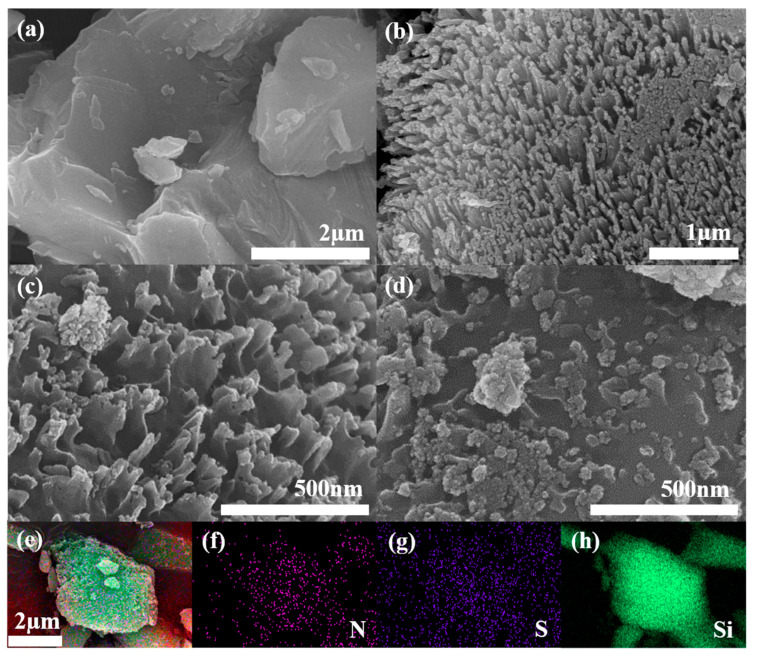
Field-emission scanning electron microscopy (FE-SEM) images of (**a**) micron silicon (mSi), (**b**) porous micron silicon (por-mSi), (**c**) polystyrene-sulfonate-grafted porous micron silicon (por-mSi@PSS), and (**d**) por-mSi@PSS anode coated with polyaniline (por-mSi@PSS/PANI) and energy-dispersive X-ray spectroscopy (EDS) elemental mapping of por-mSi@PSS@PANI for (**e**) full image, (**f**) N, (**g**) S, and (**h**) Si elements.

As can be seen from FE-SEM images (Figure 1a–d), the mSi particles (Figure 1a) are smooth and had an average diameter of 5 μm. In contrast, the por-mSi particles exhibited a porous structure with Si nanorod bundles generated by Ag-assisted chemical etching. The porosity of the por-mSi@PSS was maintained even after PSS grafting due to the formation of an ultrathin carbonaceous layer on the por-mSi surface by a one-pot pyrolysis process at a temperature below 400 °C. After an additional coating process, the por-mSi@PSS/PANI samples became blunt, with the PANI layer covering the Si surface. Nevertheless, from some of the porous structures on the surface, it was possible to confirm that the Si inside maintained its porous structure. The Si composites with double-layer coatings suppressed excessive volume expansion and improved electrical conductivity [37]. Figure 1e–h show the EDS elemental mapping images of por-mSi@PSS/PANI. Compared to por-mSi@PSS without N (Figure S1), the uniform distribution of N suggests that PANI was successfully decorated on the Si surface.

**Figure 2:**
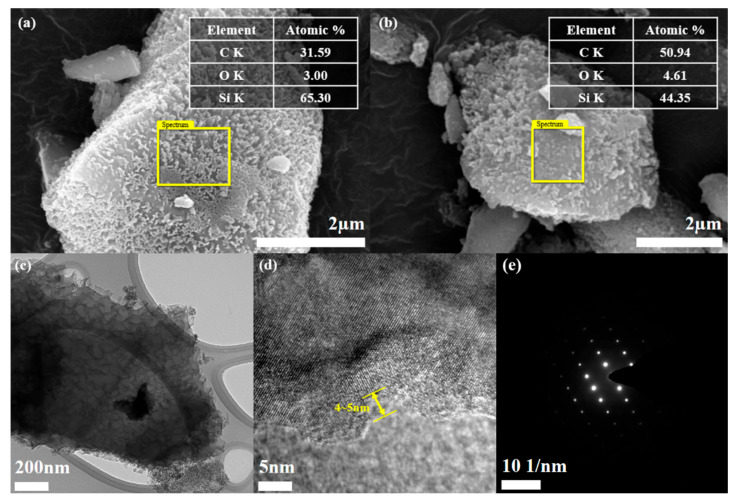
SEM images and elemental composition data of por-mSi@PSS (**a**), and por-mSi@PSS/PANI (**b**), TEM image (**c**), HR-TEM (**d**), and SAED image (**e**) of por-mSi@PSS.

The double-layer formed by both PSS pyrolysis grafting and PANI coating was confirmed by EDS analysis (Figure 2a,b). The presence of the coating layer was confirmed by the C content measurements, which were 31.59 and 50.94 at.% for por-mSi@PSS and por-mSi@PSS/PANI, respectively. In general, EDS provides a rapid quantitative analysis of elemental composition with a sampling depth of 1–2 μm [38]. However, given that the sample diameter is about 5 μm, the result can be reasonably interpreted as representing the C content present near the surface rather than the entire sample. Moreover, it is noteworthy that the high carbon content could mainly originate from the carbon tape used to anchor the sample for SEM-EDS measurements. Thus, this analysis only confirmed the successful PANI coating based on the relative increase in C content. As shown in Figure S2, the TEM analysis confirmed that the thickness of the PANI coating layer is less than 100 nm. For a more detailed observation of the ultrathin carbonaceous layer, transmission electron microscopy (TEM) and HR-TEM were performed. The TEM image (Figure 2c) of por-mSi@PSS shows that the sample had a porous structure. The HR-TEM analysis confirmed that the thickness of the PSS grafting layer was approximately 4–5 nm (Figure 2d). The selected area electron diffraction (SAED) pattern in (Figure 2e) showed that the crystal structure of the pristine por-mSi did not change, even after the grafting process. The porous nanorod structure formed by the MACE method relieved the mechanical stress caused by the volume expansion of Si during the charging and discharging process [39,40]. In addition, the thin and uniform coatings on the surface effectively prevented direct contact between the electrolyte and Si, providing additional mechanical reinforcement. Consequently, the carbonaceous coating layer formed through pyrolysis grafting enhanced the electrochemical performance by modifying the interfacial properties of the Si-based anode.

**Figure 3:**
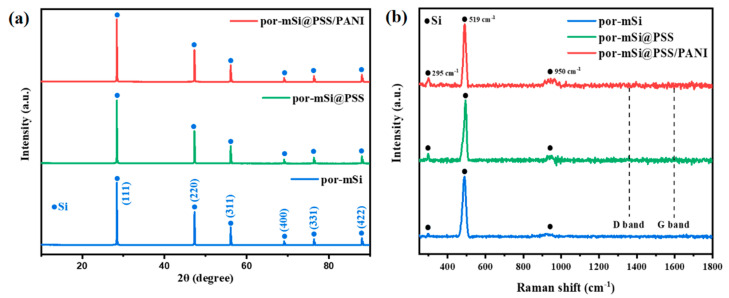
(**a**) X-ray diffraction (XRD) spectra and (**b**) Raman spectra of por-mSi, por-mSi@PSS, and por-mSi@PSS/PANI.

XRD and Raman spectroscopy were performed to analyze the properties of the prepared samples. Figure 3a shows the XRD patterns of the por-mSi, por-mSi@PSS, and por-mSi@PSS/PANI samples. All the samples exhibited the XRD peaks of crystalline Si at 28.5°, 47.3°, 56.1°, 69.1°, 76.4°, and 88.1° 2θ, corresponding to the planes (111, 220), (331), (400), and (422) of cubic Si (JCPDS Card No. 27-1402), respectively [41]. This is consistent with the crystalline structure of Si observed in the HR-TEM images, supporting the idea that only interfacial modification was performed without changing the Si structures. The Raman spectra (Figure 3b) exhibited three characteristic peaks for all the samples. The small peaks observed near 295 cm^−1^ were assigned to Si oxides associated with two transverse acoustic phonon (2TA) modes. The strong peaks at 519 cm^−1^ were attributed to the transverse optical (TO) oscillation mode of crystalline Si, while the broad peaks at 950 cm^−1^ were ascribed to the amorphous Si–Si elongation mode [42,43]. C species peaks, including the D and G bands, were not observed in the XRD and Raman spectra, strongly indicating the low C content and amorphous nature of the ultrathin phenyl grafts [44].

**Figure 4:**
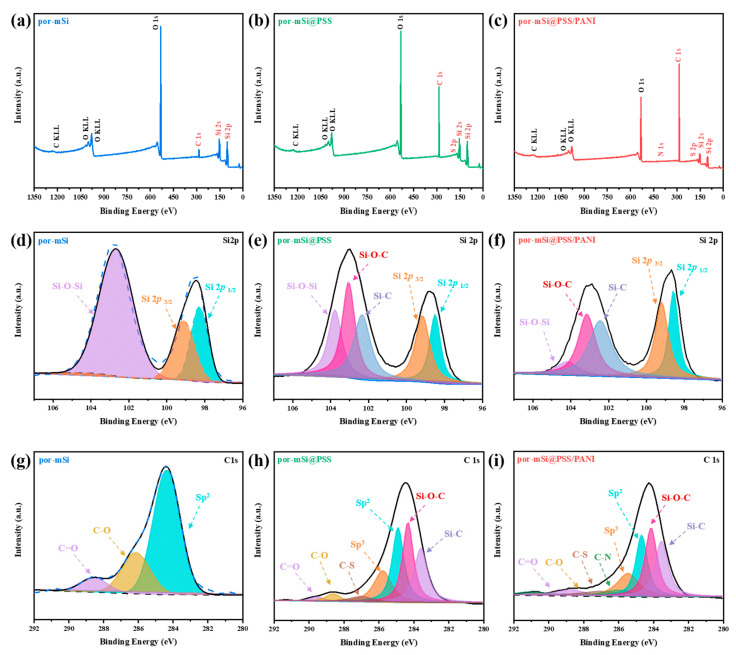
Deconvoluted X-ray photoelectron spectroscopy (XPS) spectra for por-mSi, por-mSi@PSS and por-mSi@PSS/PANI samples. (**a**–**c**) Survey scan, (**d**–**f**) Si 2p core spectra, and (**g**–**i**) C 1s core spectra.

XPS was performed to characterize the surfaces of the prepared samples. The survey scans (Figure 4a–c) of por-mSi, por-mSi@PSS, and por-mSi@PSS/PANI showed clear peaks at 284, 532, and 99 eV for C 1s, O 1s, and Si 2p, respectively. After PSS pyrolysis grafting, the C 1s peak increased, as shown in (Figure 4b). In addition, the increase in the C 1s peak after the additional PANI coating (Figure 4c) suggest that abundant C species were present on the Si surface [45]. The high-resolution core-level spectrum of Si 2p confirmed the oxidation state of the Si composite. In the core-level spectra of the samples (Figure 4d–f), the Si 2p spectrum was divided into five components of Si 2p 1/2 at 98.3 eV, Si 2p 3/2 at 99.4 eV, Si–C at 102.3 eV, Si–O–C at 103.1 eV, and Si–O–Si at 103.5 eV [46,47]. During pyrolysis grafting, Si–C and Si–O–C species were formed in the por-mSi@PSS and por-mSi@PSS/PANI samples, respectively. In particular, the Si surface was significantly protected in por-mSi@PSS/PANI, as indicated by the negligible levels of the Si–O–Si peaks (Figure 4f). In contrast, the Si 2p core spectrum of por-mSi (Figure 4d) showed a dominant peak at 103.5 eV, indicating that the Si surface was more prone to oxidation. These results suggested that the formation of Si–O–C and Si–C covalent bonds improved the stability of Si. In addition, the C 1s core spectra (Figure 4h,i) of the prepared Si composite were resolved into sp^2^- and sp^3^-hybridized states of C–C bonds at 284.5 and 285.7 eV, respectively, with peaks corresponding to C–N and C–S bonds at 286.1 and 287.2 eV, respectively. In contrast, the C 1s spectrum of por-mSi predominantly showed sp^2^ hybridization peaks, with only C=O and C–O peaks [48]. The C peak observed in Figure 4g corresponds to impurities, commonly referred to as ‘adventitious carbon’ (AdC). It is well established that surface contamination tends to increase with longer storage times, and even storage under vacuum conditions does not completely eliminate this issue [49]. In particular, the porous nature of the por-mSi sample makes it highly susceptible to contamination from the surrounding environment due to its high specific surface area. On the other hand, Figure 4h,i show a significant reduction in AdC signals, and Si-C and Si-O-C peaks were observed, indicating the presence of the PSS grafting layer. Since the thickness of the PSS layer (4–5 nm) is not thick enough to interfere with the XPS analysis of the underlying Si surface, it is reasonable to interpret that the formation of AdC is suppressed after grafting.

**Figure 5:**
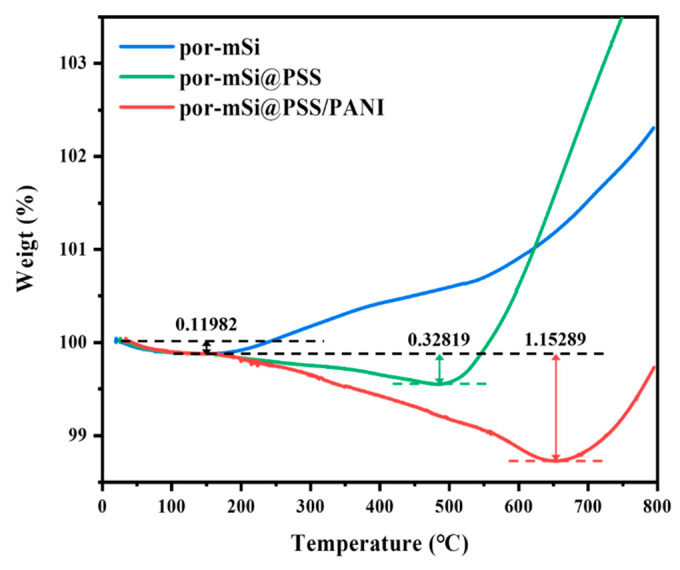
Thermogravimetric analysis (TGA) of por-mSi, por-mSi@PSS, and por-mSi@PSS/PANI.

The thermogravimetric analysis (TGA) shown in Figure 5 was performed to determine quantitatively the carbon content present in the prepared samples. The initial weight loss of 0.12 wt% (up to 150 °C), which occurs equally in all samples, is due to the evaporation of physically absorbed water molecules and volatile organic matter. For por-mSi without polymer grafting, the sample weight starts to increase around 200 °C, attributed to the oxidation of porous silicon by residual oxygens that remains despite N_2_ purging. On the other hand, por-mSi@PSS and por-mSi@PSS/PANI exhibit weight losses of 0.33 wt% at 490 °C and 1.15 wt% at 660 °C, respectively, due to the decomposition of PSS fragment and PANI under inert N_2_ conditions. After the complete decomposition of the protective surface layer, the Si surface starts to oxidize further due to the abundant oxygen species derived from the sulfone group of PSS, resulting in a rapid increase in the TGA curves around 500–600 °C. These results strongly support that the PSS-grafted, PANI-coated double layer present in trace amount on the composite surface strengthens the resistance of silicon anode to oxidation.

The thermolytic grafting mechanism illustrated in Scheme S2 involves the addition of styrene-based radical fragments via a radical mechanism. PSS, an amorphous linear polymer synthesized through a free radical polymerization process, undergoes thermal decomposition to produce polymeric radicals (including styrene monomers and oligomers) through depolymerization process. Phenyl radical intermediates are relatively stable due to the resonant electron delocalization within aromatic rings. As a result, the phenyl radicals generated during the thermal decomposition of PSS can persist sufficiently to interact with Si-H groups or free radicals on the surface of por-mSi [50]. In addition, the para-substituted SO_3_^−^ group enhances this stability by donating lone pair electrons to the phenyl ring through resonance, ensuring that the carbocation species at the benzyl site remain more stable throughout the pyrolysis process [51]. Consequently, phenyl radicals derived from PSS can efficiently convert Si-O-Si/Si-Si bonds into Si-O-C/Si-C bonds. The increased proportion of Si-O-C and Si-C bonds at the Si interface can impart stronger chemical and mechanical resistance against corrosive electrochemical environments and large volume changes in Si active materials.

### 3.2. Electrochemical Properties and Performance of pSi Composites

**Figure 6:**
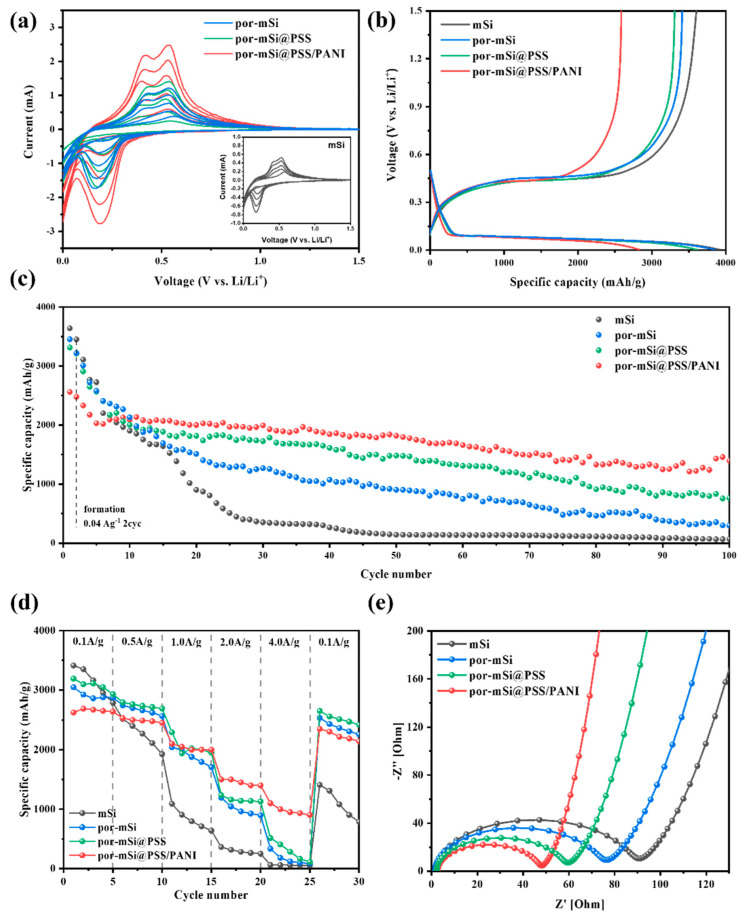
(**a**) Cyclic voltammetry of prepared anodes from 1st to 5th cycle. (**b**) Galvanostatic charge–discharge profiles for the first cycles of mSi, por-mSi, por-mSi@PSS, and por-mSi@PSS/PANI at a current density of 0.04 A g^−1^. (**c**) Cycling performance of mSi, por-mSi, por-mSi@PSS, and por-mSi@PSS/PANI at 0.1 A g^−1^. (**d**) Rate capability of prepared samples. (**e**) Nyquist plot of mSi, por-mSi, and por-mSi@PSS/PANI samples before cycling.

Figure 6a shows the CV curves of Si-based anodes from the first to the fifth cycle in a potential window of 0.01–1.5 V at a scan rate of 0.2 mV s^−1^. The peak at 0.19 V corresponded to the transition of crystalline Si into the amorphous Li_x_Si phase during lithiation [52,53]. On the other hand, the two peaks at 0.42 V and 0.53 V corresponded to the extraction process of Li^+^ from Li_x_Si during delithiation [54,55]. All samples exhibited similar CV curves, indicating that the grafted PSS and PANI coatings did not interfere with the lithiation and delithiation processes of the Si active material. The increase in the redox current with an increasing number of CV cycles was attributed to the intercalation/extraction of more Li^+^ ions into the active material [56]. This process occurred when the Si electrode and electrolyte interface were activated during the initial cycling (formation process) [57,58]. This trend is consistent with the higher redox currents observed for por-mSi, por-mSi@PSS, and por-mSi@PSS/PANI, which had more active sites than mSi.

The charge–discharge profiles for the first cycles of mSi, por-mSi, por-mSi@PSS, and por-mSi@PSS/PANI samples are shown in Figure 6b. The initial charging capacity of por-mSi@PSS/PANI was 2826 mAh g^−1^, with a discharging capacity of 2561 mAh g^−1^, and a CE of 90.6%. The irreversible capacity loss of 9.4% is mainly due to the cumulative effect of SEI layer formation during cycling and the irreversible insertion of Li^+^ ions into the Si active material [59]. Likewise, mSi, por-mSi, and por-mSi@PSS exhibited initial charging capacities of 3981, 3744, and 3612 mAh g^−1^, respectively, with corresponding discharging capacities of 3639, 3452, and 3313 mAh g^−1^, and CE values of 91.4%, 88.3%, and 91.7%, respectively. The relatively lower initial CE of por-mSi compared to mSi may be due to its higher specific surface area, consequently increasing electrolyte consumption and irreversible reactions during SEI formation [60].

Figure 6c shows the cycling performance of mSi, por-mSi, por-mSi@PSS, and por-mSi@PSS/PANI anodes. The Coulombic efficiencies (CE) of por-mSi, por-mSi@PSS, and por-mSi@PSS/PANI were stabilized by reaching ~95% after the initial 10 cycles, including two formation cycles, which were then tested at a current density of 0.1 A g^−1^. The pristine mSi also exhibited a CE of ~95% after 25 cycles. After the stabilization step, por-mSi@PSS/PANI exhibited the highest capacity of 1820 mAh g^−1^ at 0.1 A g^−1^ after 50 cycles, with a capacity retention rate of 89.5%. On the other hand, the capacity retention rates of por-Si and por-mSi@PSS were 35% and 57.4%, respectively. Specifically, mSi exhibited a very low capacity retention of ~5% after 50 cycles, likely due to mechanical stress, fracturing, and separation from the electrode caused by the rapid volume expansion of mSi during the charging and discharging process [61]. After 100 cycles, it is noteworthy that por-mSi@PSS/PANI still showed a capacity retention rate of 54.6%, while the other samples are showed only 1.9, 8.6, and 23.1%, respectively. This highlights the significant impact that modifications to the interfacial properties of Si with polymers can have on the cycling performance.

As shown in TGA of PSS (Figure S4a), the PSS polymer undergoes three distinct weight loss stages. The initial weight loss of 11.048% (up to 150 °C) is due to the evaporation of moisture and volatile organic compounds. Subsequently, the decomposition of PSS fragments in the range of 350–450 °C accounts for an additional weight loss of 11.657%, and the polymer residues are completely carbonized beyond 500 °C. The cycling performance of por-mSi@PSS, grafted at 360 and 400 °C, is shown in Figure S4b. Styrene-based polymers typically decompose and volatilize into small molecules at high temperatures more than 400 °C. This rapid pyrolysis can lead to the uneven grafting and oxidation of silicon. In contrast, at the relatively lower temperature of 360 °C, a larger fraction of PSS fragments is uniformly grafted onto por-mSi, forming a thin phenyl-carbon layer. As a result, por-mSi@PSS grafted at 360 °C exhibits a higher cycling performance compared to that grafted at 400 °C. The comparative cycling performance of por-mSi@PSS grafted at 360 and 400 °C indicates the more effectiveness of the thermolytic grafting process at the lower temperature of 360 °C. In contrast, por-mSi@PSS grafted at 400 °C shows a reduced performance, probably due to the increased volatilization, which inhibits the effective grafting of PSS fragments onto the Si surface.

Rate capability tests were performed to evaluate the high-power performance and capacity recovery characteristics of the Si-composite anode (Figure 6d). All prepared samples were tested with five cycles at each current density, ranging from 0.1 to 4.0 A g^−1^, and then again at 0.1 A g^−1^. The initial discharge capacities of mSi, por-mSi, por-mSi@PSS, and por-mSi@PSS/PANI were 3412, 3046, 3192, and 2625 mAh g^−1^, respectively, following the same trends in initial capacities as observed in cycling tests. Despite its lower initial discharge capacity due to the additional polymer coating, por-mSi@PSS/PANI achieved an impressive capacity of 1077 mAh g^−1^ at a high current density of 4 A g^−1^ and recovered 89% of its initial capacity when returned to 0.1 A g^−1^. In comparison, the initial capacity recoveries for mSi, por-mSi, and por-mSi@PSS were 41%, 74%, and 75%, respectively.

Impedance measurements were performed using EIS to assess the interfacial resistance of the prepared Si-composite anode material. Figure 6e shows the impedance spectra of the mSi, por-mSi@PSS, and por-mSi@PSS/PANI samples measured at an amplitude of 5 mV in the frequency range of 0.01 to 1 MHz. The diameter of the semicircle in the Nyquist plot indicates that impedance was influenced by the surface shape of the electrode material. The semicircle in the high-frequency domain is related to the formation of an SEI, whereas the oblique line represents Li-ion diffusion in the electrode material [62,63]. In the analysis of the EIS data, the por-mSi@PSS/PANI semicircle was the smallest of all samples, which contributed to the stable interfacial formation of the amorphous layer, and the additional coating of conductive polyaniline could reduce the interfacial resistance [64,65,66].

The Nyquist plots of all anode cells were fitted to the equivalent circuits shown in Figure S3. R_e_, R_SEI+ct_, and W represent the electrolyte impedance, the sum of SEI layer resistance and charge transfer resistance, and the Warburg impedance, respectively. As shown in the table in Figure S3, the R_SEI+ct_ value of por-mSi@PSS/PANI was the lowest among the prepared samples (41.574 Ω). This suggests that the ultrathin phenyl grafts contributed to the formation of a stable SEI layer, and that the conductive PANI coating promoted fast charge transfer of Li^+^ ions. The smallest Warburg impedance value (9.509 mΩ ^s−1/2^) also clearly supports the above claim.

**Figure 7:**
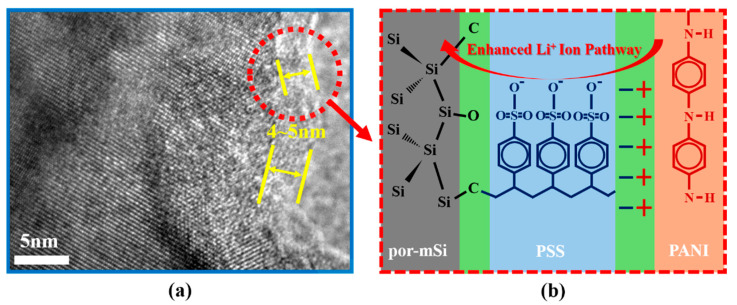
Schematic of Li+ ion storage mechanism of por-mSi@PSS/PANI. (**a**) Si and styrene C fragments formed a solid Si–C/Si–O–C bond (PSS layer of 4–5 nm shown in HR-TEM images), and (**b**) a conductive polymer layer attached by electrostatic attraction contributed to the improved transfer of Li+ ions and electrons.

Figure 7 illustrates the Li+ storage mechanism of the por-mSi@PSS/PANI samples. Pristine mSi underwent significant mechanical stress owing to excessive volume expansion during charging and discharging cycles. This repeated cycling led to cracking, grinding, and eventual peeling off of the active material from the electrode. Furthermore, the repeated formation of new SEI layers on the exposed Si surface resulted in a rapid decrease in capacity. Additionally, por-mSi experienced unintended irreversible reactions owing to the direct contact between the active material surface and the electrolyte. In contrast, the por-mSi@PSS anode benefited from effective protection by the grafted PSS through the Si–O–C and Si–C linkages. Specifically, strong Si–C covalent bonds suppressed the volume expansion of Si, preventing damage to the active material and minimizing excessive SEI layer formation. The Si–O–C bonds, along with the sulfone group-containing styrene C fragments, promoted the adsorption of Li+ ions because of the high electronegativity of oxygen and sulfur. As a result, the grafted anodes exhibited a higher reversible capacity and more stable cycling performance than their non-grafted counterparts (mSi and por-mSi). Moreover, positively charged conductive polyaniline polymers were electrostatically attached to the negatively charged por-mSi@PSS surface, improving the conductivity of Si while providing additional protective layers.

Table S1 compares and summarizes the recently reported electrochemical performance of Si–C-based anode materials. Compared to other studies, por-mSi@PSS/PANI demonstrated comparable or superior cycling performance and excellent rate capability. Notably, it exhibited high stability despite the use of micron-sized bulk Si as opposed to highly purified and costly nanosized Si. In addition, most other anode studies were performed under complex and uneconomical synthesis conditions (e.g., high temperature, high pressure, plasma), with a relatively high proportion of carbon in the active material (40~60 wt%); however, our approach is distinguished by its relatively simple and cost-effective synthesis process. Furthermore, our materials have an exceptionally high Si content (98.85 wt%), distinguishing it from that of other recent studies.

## 4. Conclusions

In summary, por-mSi@PSS/PANI composites synthesized by facile one-pot pyrolysis and conductive PANI coating demonstrated excellent electrochemical performance as Li storage anodes. Specifically, the por-mSi@PSS/PANI achieved an excellent reversible capacity of 1820 mAh g^−1^ at 0.1 A g^−1^ after 50 cycles, along with a high capacity of 1077 mAh g^−1^ at 4 A g^−1^ and an impressive capacity retention of 89.5% when the current density was returned to 0.1 A g^−1^. This outstanding performance can be attributed to the ultrathin phenyl grafts and conductive PANI coatings on the Si surface, which effectively suppressed volume expansion and enhanced Li-ion transport. This paper presents a simple interfacial engineering strategy for the development of advanced LIB anodes.

## Data Availability

The original contributions presented in the study are included in the article/ Supplementary Materials, and further inquiries can be directed to the corresponding author.

## Supplementary Materials

The following supporting information can be downloaded from https://www.mdpi.com/article/10.3390/polym16243544/s1. Scheme S1. Syntheses of por-mSi, por-mSi@PSS, and por-mSi@PSS/PANI. Scheme S2. Proposed thermolytic grafting mechanism for the bonding of styrenic carbon at the interface of porous silicon composites. Figure S1. EDS elemental mapping of por-mSi@PSS. Figure S2. TEM image of por-mSi@PSS/PANI. Figure S3. EIS data of the as-synthesized samples. Figure S4. TGA of PSS and Cycling performance of por-mSi@PSS (360, 400 °C) samples. Table S1. Summary of the literature on the electrochemical performance of Si-C based anode materials. References [67,68,69,70,71,72,73,74,75,76,77,78] are cited in the supplementary materials.

## References

[1] Chen Y., Kang Y., Zhao Y., Wang L., Liu J., Li Y., Liang Z., He X., Li X., Tavajohi N. (2021). A review of lithium-ion battery safety concerns: The issues, strategies, and testing standards. J. Energy Chem..

[2] Nitta N., Wu F., Lee J.T., Yushin G. (2015). Li-ion battery materials: Present and future. Mater. Today.

[3] Choi S., Wang G. (2018). Advanced Lithium-Ion Batteries for Practical Applications: Technology, development, and future perspectives. Adv. Mater. Technol..

[4] Wakihara M. (2001). Recent developments in lithium ion batteries. Mater. Sci. Eng. R Rep..

[5] Hossain M.H., Chowdhury M.A., Hossain N., Islam M.A., Mobarak M.H. (2023). Advances of lithium-ion batteries anode materials—A review. Chem. Eng. J. Adv..

[6] Selvi K.T., Mangai K.A., Lett J.A., Fatimah I., Sagadevan S. (2024). Exploring the electrode materials for high-performance lithium-ion batteries for energy storage application. J. Energy Storage.

[7] Xu J., Cai X., Cai S., Shao Y., Hu C., Lu S., Ding S. (2023). High-Energy Lithium-Ion Batteries: Recent Progress and a Promising Future in Applications. Energy Environ. Mater..

[8] Cao D., Sun X., Li Q., Natan A., Xiang P., Zhu H. (2020). Lithium Dendrite in All-Solid-State Batteries: Growth Mechanisms, Suppression Strategies, and Characterizations. Matter.

[9] Rahardian S., Budiman B.A., Sambegoro P.L., Nurprasetio I.P. Review of Solid-State Battery Technology Progress. Proceedings of the 2019 6th International Conference on Electric Vehicular Technology (ICEVT).

[10] Janek J., Zeier W.G. (2023). Challenges in speeding up solid-state battery development. Nat. Energy.

[11] Li Y., Li Q., Chai J., Wang Y., Du J., Chen Z., Rui Y., Jiang L., Tang B. (2023). Si-based Anode Lithium-Ion Batteries: A Comprehensive Review of Recent Progress. ACS Mater. Lett..

[12] Sun L., Liu Y., Shao R., Wu J., Jiang R., Jin Z. (2022). Recent progress and future perspective on practical silicon anode-based lithium ion batteries. Energy Storage Mater..

[13] Feng K., Li M., Liu W., Kashkooli A.G., Xiao X., Cai M., Chen Z. (2018). Silicon-Based Anodes for Lithium-Ion Batteries: From Fundamentals to Practical Applications. Small.

[14] Zhang C., Wang F., Han J., Bai S., Tan J., Liu J., Li F. (2021). Challenges and Recent Progress on Silicon-Based Anode Materials for Next-Generation Lithium-Ion Batteries. Small Struct..

[15] Jerliu B., Hüger E., Dörrer L., Seidlhofer B.K., Steitz R., Oberst V., Geckle U., Bruns M., Schmidt H. (2014). Volume Expansion during Lithiation of Amorphous Silicon Thin Film Electrodes Studied by In-Operando Neutron Reflectometry. J. Phys. Chem. C.

[16] Schmidt H., Jerliu B., Hüger E., Stahn J. (2020). Volume expansion of amorphous silicon electrodes during potentiostatic lithiation of Li-ion batteries. Electrochem. Commun..

[17] Li L., Deng Y., Hu K., Xu B., Wang N., Bai Z., Xu X., Yang J. (2023). Nanostructure designing and hybridizing of high-capacity silicon-based anode for lithium-ion batteries. Prog. Nat. Sci. Mater. Int..

[18] Loewenich M., Orthner H., Wollny P., Wlokas I., Bade S., Lyubina J., Wiggers H. (2024). Synthesis and upscaling of silicon nanoparticles for lithium-ion batteries in a hot-wall reactor. J. Alloys Compd..

[19] Raić M., Mikac L., Marić I., Štefanić G., Škrabić M., Gotić M., Ivanda M. (2020). Nanostructured Silicon as Potential Anode Material for Li-Ion Batteries. Molecules.

[20] Qi C., Li S., Yang Z., Xiao Z., Zhao L., Yang F., Ning G., Ma X., Wang C., Xu J. (2022). Suitable thickness of carbon coating layers for silicon anode. Carbon.

[21] Yu J., Yang J., Feng X., Jia H., Wang J., Lu W. (2014). Uniform Carbon Coating on Silicon Nanoparticles by Dynamic CVD Process for Electrochemical Lithium Storage. Ind. Eng. Chem. Res..

[22] Li X., Zhang M., Yuan S., Lu C. (2020). Research Progress of Silicon/Carbon Anode Materials for Lithium-Ion Batteries: Structure Design and Synthesis Method. ChemElectroChem.

[23] Dou F., Shi L., Chen G., Zhang D. (2019). Silicon/Carbon Composite Anode Materials for Lithium-Ion Batteries. Electrochem. Energy Rev..

[24] Shen X., Tian Z., Fan R., Shao L., Zhang D., Cao G., Kou L., Bai Y. (2018). Research progress on silicon/carbon composite anode materials for lithium-ion battery. J. Energy Chem..

[25] Huang Z., Farahmandjou M., Marlton F., Guo X., Gao H., Sun B., Wang G. (2024). Surface and structure engineering of MXenes for rechargeable batteries beyond lithium. J. Mater..

[26] Yang Q., Wang Z., Xia Y., Wu G., Chen C., Wang J., Rao P., Dong A. (2020). Facile electrostatic assembly of Si@MXene superstructures for enhanced lithium-ion storage. J. Colloid Interface Sci..

[27] Zhang F., Jia Z., Wang C., Feng A., Wang K., Hou T., Liu J., Zhang Y., Wu G. (2020). Sandwich-like silicon/Ti3C2Tx MXene composite by electrostatic self-assembly for high performance lithium ion battery. Energy.

[28] Xu Y., Sun X., Wei C., Liang G. (2020). Self-assembly by electrostatic attraction to encapsulate Si in N-rich graphene for high performance lithium-ion batteries. J. Electroanal. Chem..

[29] Salem A.M.S., Harraz F.A., El-Sheikh S.M., Ismat Shah S. (2020). Novel Si nanostructures via Ag-assisted chemical etching route on single and polycrystalline substrates. Mater. Sci. Eng. B.

[30] Xuan Tran M., Woo J.-Y., Nguyen T.-A., Lee S.-W., Kee Lee J. (2020). Thermolytically grafted silicon particles with ultrathin carbonaceous coating rich of phenyl moieties as lithium-storage anode material. Chem. Eng. J..

[31] Tran M.X., Nguyen T.-A., Lee J.K., Lee S.-W. (2023). Porous silicon covalently-grafted with chloro-styrenic carbons for fast Li^+^ diffusion and durable lithium-storage capability. J. Power Sources.

[32] Naikwade M.B., Lee Y.C., Salunkhe T.T., Kim I.T., Nguyen T.-A., Kadam A., Lee S.-W. (2024). Enhanced Lithium Storage in Micro-Si-Based Anode Materials through Low-Temperature Interface Engineering with an Ultrathin Phenolic Interlayer. ACS Appl. Energy Mater..

[33] Lee K., Kim T.-H. (2018). Poly(aniline-co-anthranilic acid) as an electrically conductive and mechanically stable binder for high-performance silicon anodes. Electrochim. Acta.

[34] Feng M., Tian J., Xie H., Kang Y., Shan Z. (2015). Nano-silicon/polyaniline composites with an enhanced reversible capacity as anode materials for lithium ion batteries. J. Solid State Electrochem..

[35] Pera D.M., Costa I., Serra F., Gaspar G., Lobato K., Serra J.M., Silva J.A. (2023). Development of a metal-assisted chemical etching method to improve light-capture in monocrystalline silicon solar cells. Sol. Energy Mater. Sol. Cells.

[36] Wang J., Joo J., Kennard R.M., Lee S.-W., Sailor M.J. (2016). Thermolytic Grafting of Polystyrene to Porous Silicon. Chem. Mater..

[37] Tian Y., Li Y., Xiao P., Zhou P., Fang Z., Li Y. (2023). Carbon coating optimization on porous silicon as high-performance anode material via fluidized bed chemical vapor deposition. J. Alloys Compd..

[38] Shaban S.E., Ibrahiem N.M., El-mongy S.A., Elshereafy E.E. (2013). Validation of scanning electron microscope (SEM), energy dispersive X-ray (EDX) and gamma spectrometry to verify source nuclear material for safeguards purposes. J. Radioanal. Nucl. Chem..

[39] Dai F., Yi R., Yang H., Zhao Y., Luo L., Gordin M.L., Sohn H., Chen S., Wang C., Zhang S. (2019). Minimized Volume Expansion in Hierarchical Porous Silicon upon Lithiation. ACS Appl. Mater. Interfaces.

[40] Maroni F., Spreafico M., Schönecker A., Wohlfahrt-Mehrens M., Marinaro M. (2022). Near-Zero Volume Expansion Nanoporous Silicon as Anode for Li-ion Batteries. J. Electrochem. Soc..

[41] Wahab R., Ahmad N., Alam M. (2020). Silicon nanoparticles: A new and enhanced operational material for nitrophenol sensing. J. Mater. Sci. Mater. Electron..

[42] Zhang S.L., Wang X., Ho K.S., Li J., Diao P., Cai S. (1994). Raman spectra in a broad frequency region of p- type porous silicon. J. Appl. Phys..

[43] Du F.-H., Wang K.-X., Fu W., Gao P.-F., Wang J.-F., Yang J., Chen J.-S. (2013). A graphene-wrapped silver–porous silicon composite with enhanced electrochemical performance for lithium-ion batteries. J. Mater. Chem. A.

[44] Guo Z.P., Milin E., Wang J.Z., Chen J., Liu H.K. (2005). Silicon/Disordered Carbon Nanocomposites for Lithium-Ion Battery Anodes. J. Electrochem. Soc..

[45] Khung Y.L., Ngalim S.H., Scaccabarozzi A., Narducci D. (2015). Formation of stable Si–O–C submonolayers on hydrogen-terminated silicon(111) under low-temperature conditions. Beilstein J. Nanotechnol..

[46] Avila A., Montero I., Galán L., Ripalda J.M., Levy R. (2001). Behavior of oxygen doped SiC thin films: An x-ray photoelectron spectroscopy study. J. Appl. Phys..

[47] George V.C., Das A., Roy M., Dua A.K., Raj P., Zahn D.R.T. (2002). Bias enhanced deposition of highly oriented β-SiC thin films using low pressure hot filament chemical vapour deposition technique. Thin Solid Film..

[48] Zhou X., Yin Y.-X., Wan L.-J., Guo Y.-G. (2012). Facile synthesis of silicon nanoparticles inserted into graphene sheets as improved anode materials for lithium-ion batteries. Chem. Commun..

[49] Greczynski G., Hultman L. (2020). X-ray photoelectron spectroscopy: Towards reliable binding energy referencing. Prog. Mater. Sci..

[50] Wang D., Buriak J.M. (2006). Trapping Silicon Surface-Based Radicals. Langmuir.

[51] Valencia D. (2017). Elucidating the structure of light absorbing styrene carbocation species formed within zeolites. Phys. Chem. Chem. Phys..

[52] Fan Y., Zhang Q., Xiao Q., Wang X., Huang K. (2013). High performance lithium ion battery anodes based on carbon nanotube-silicon core-shell nanowires with controlled morphology. Carbon.

[53] Wang W., Kumta P.N. (2010). Nanostructured hybrid silicon/carbon nanotube heterostructures: Reversible high-capacity lithium-ion anodes. ACS Nano.

[54] Suresh S., Wu Z.P., Bartolucci S.F., Basu S., Mukherjee R., Gupta T., Hundekar P., Shi Y., Lu T.M., Koratkar N. (2017). Protecting Silicon Film Anodes in Lithium-Ion Batteries Using an Atomically Thin Graphene Drape. ACS Nano.

[55] Liu J., Kopold P., van Aken P.A., Maier J., Yu Y. (2015). Energy Storage Materials from Nature through Nanotechnology: A Sustainable Route from Reed Plants to a Silicon Anode for Lithium-Ion Batteries. Angew. Chem. Int. Ed. Engl..

[56] Yang X., Wen Z., Xu X., Lin B., Huang S. (2007). Nanosized silicon-based composite derived by in situ mechanochemical reduction for lithium ion batteries. J. Power Sources.

[57] Ashuri M., He Q., Liu Y., Shaw L.L. (2020). Investigation towards scalable processing of silicon/graphite nanocomposite anodes with good cycle stability and specific capacity. Nano Mater. Sci..

[58] Schroder K.W., Celio H., Webb L.J., Stevenson K.J. (2012). Examining Solid Electrolyte Interphase Formation on Crystalline Silicon Electrodes: Influence of Electrochemical Preparation and Ambient Exposure Conditions. J. Phys. Chem. C.

[59] Yu Y., Gu L., Zhu C., Tsukimoto S., van Aken P.A., Maier J. (2010). Reversible Storage of Lithium in Silver-Coated Three-Dimensional Macroporous Silicon. Adv. Mater..

[60] Bärmann P., Mohrhardt M., Frerichs J.E., Helling M., Kolesnikov A., Klabunde S., Nowak S., Hansen M.R., Winter M., Placke T. (2021). Mechanistic Insights into the Pre-Lithiation of Silicon/Graphite Negative Electrodes in “Dry State” and After Electrolyte Addition Using Passivated Lithium Metal Powder. Adv. Energy Mater..

[61] Szczech J.R., Jin S. (2011). Nanostructured silicon for high capacity lithium battery anodes. Energy Environ. Sci..

[62] Lozhkina D.A., Rumyantsev A.M., Astrova E.V. (2020). Impedance Spectroscopy of Porous Silicon and Silicon-Carbon Anodes Produced by Sintering. Semiconductors.

[63] Wang X., Zhu J., Dai H., Yu C., Wei X. (2023). Impedance Investigation of Silicon/Graphite Anode during Cycling. Batteries.

[64] Mou T., Wang B. (2018). Rational Surface Modification of Two-Dimensional Layered Black Phosphorus: Insights from First-Principles Calculations. ACS Omega.

[65] Mou W., Ohmura S., Hattori S., Nomura K.I., Shimojo F., Nakano A. (2012). Enhanced charge transfer by phenyl groups at a rubrene/C 60 interface. J. Chem. Phys..

[66] Nulu A., Nulu V., Moon J.S., Sohn K.Y. (2021). Unified NCNT@rGO bounded porous silicon composite as an anode material for Lithium-ion batteries. Korean J. Chem. Eng..

[67] Wang Z., Mao Z., Lai L., Okubo M., Song Y., Zhou Y., Liu X., Huang W. (2017). Sub-micron silicon/pyrolyzed carbon@natural graphite self-assembly composite anode material for lithium-ion batteries. Chem. Eng. J..

[68] He Y., Han F., Wang F., Tao J., Wu H., Zhang F., Liu J. (2021). Optimal microstructural design of pitch-derived soft carbon shell in yolk-shell silicon/carbon composite for superior lithium storage. Electrochim. Acta.

[69] Li J.-Y., Li G., Zhang J., Yin Y.-X., Yue F.-S., Xu Q., Guo Y.-G. (2019). Rational design of robust Si/C microspheres for high-tap-density anode materials. ACS Appl. Mater. Interfaces.

[70] Yi Z., Lin N., Zhao Y., Wang W., Qian Y., Zhu Y., Qian Y. (2019). A flexible micro/nanostructured Si microsphere cross-linked by highly-elastic carbon nanotubes toward enhanced lithium ion battery anodes. Energy Storage Mater..

[71] Hsieh C.-C., Liu W.-R. (2019). Effects of nitrogen doping on Si/carbon composite anode derived from Si wastes as potential active materials for Li ion batteries. J. Alloys Compd..

[72] Chen C.-Y., Liang A.-H., Huang C.-L., Hsu T.-H., Li Y.-Y. (2020). The pitch-based silicon-carbon composites fabricated by electrospraying technique as the anode material of lithium ion battery. J. Alloys Compd..

[73] Zhang W., Fang S., Wang N., Zhang J., Shi B., Yu Z., Yang J. (2020). A compact silicon–carbon composite with an embedded structure for high cycling coulombic efficiency anode materials in lithium-ion batteries. Inorg. Chem. Front..

[74] Wang Q., Meng T., Li Y., Yang J., Huang B., Ou S., Meng C., Zhang S., Tong Y. (2021). Consecutive chemical bonds reconstructing surface structure of silicon anode for high-performance lithium-ion battery. Energy Storage Mater..

[75] Zhang Y., Wang Z., Hu K., Ren J., Yu N., Liu X., Wu G., Liu N. (2021). Anchoring silicon on the basal plane of graphite via a three-phase heterostructure for highly reversible lithium storage. Energy Storage Mater..

[76] Zhou Y., Yang Y., Hou G., Yi D., Zhou B., Chen S., Lam T.D., Yuan F., Golberg D., Wang X. (2020). Stress-relieving defects enable ultra-stable silicon anode for Li-ion storage. Nano Energy.

[77] Ren W., Zhang Z., Wang Y., Tan Q., Zhong Z., Su F. (2015). Preparation of porous silicon/carbon microspheres as high performance anode materials for lithium ion batteries. J. Mater. Chem. A.

[78] Hou Y., Li J., Wen Z., Cui S., Yuan C., Chen J. (2014). N-doped graphene/porous g-C3N4 nanosheets supported layered-MoS2 hybrid as robust anode materials for lithium-ion batteries. Nano Energy.

