# Supplementary material for: Interface Engineering of Styrenic Polymer Grafted Porous Micro-Silicon/Polyaniline Composite for Enhanced Lithium Storage Anode Materials
Source: Polymers (Basel). 2024 Dec 19;16(24):3544. doi: 10.3390/polym16243544 (PMC11679390; doi:10.3390/polym16243544)
Supplement: Supplementary file 1 [file polymers-16-03544-s001.zip › polymers-3363585-supplementary.pdf]

Supplementary Materials

for

Interface Engineering of Styrenic Polymer Grafted Porous Micro-Silicon/Polyaniline Composite for Enhanced Lithium Storage Anode Materials

Yechan Lee, Mahesh Naikwade, and Sang-Wha Lee*

Department of Chemical and Biological Engineering, Gachon University, 1342 Seongnamdaero, Su-jeong-Gu, Seongnam-Si 13120, Gyeonggi-do, Republic of Korea

* Correspondence: lswha@gachon.ac.kr

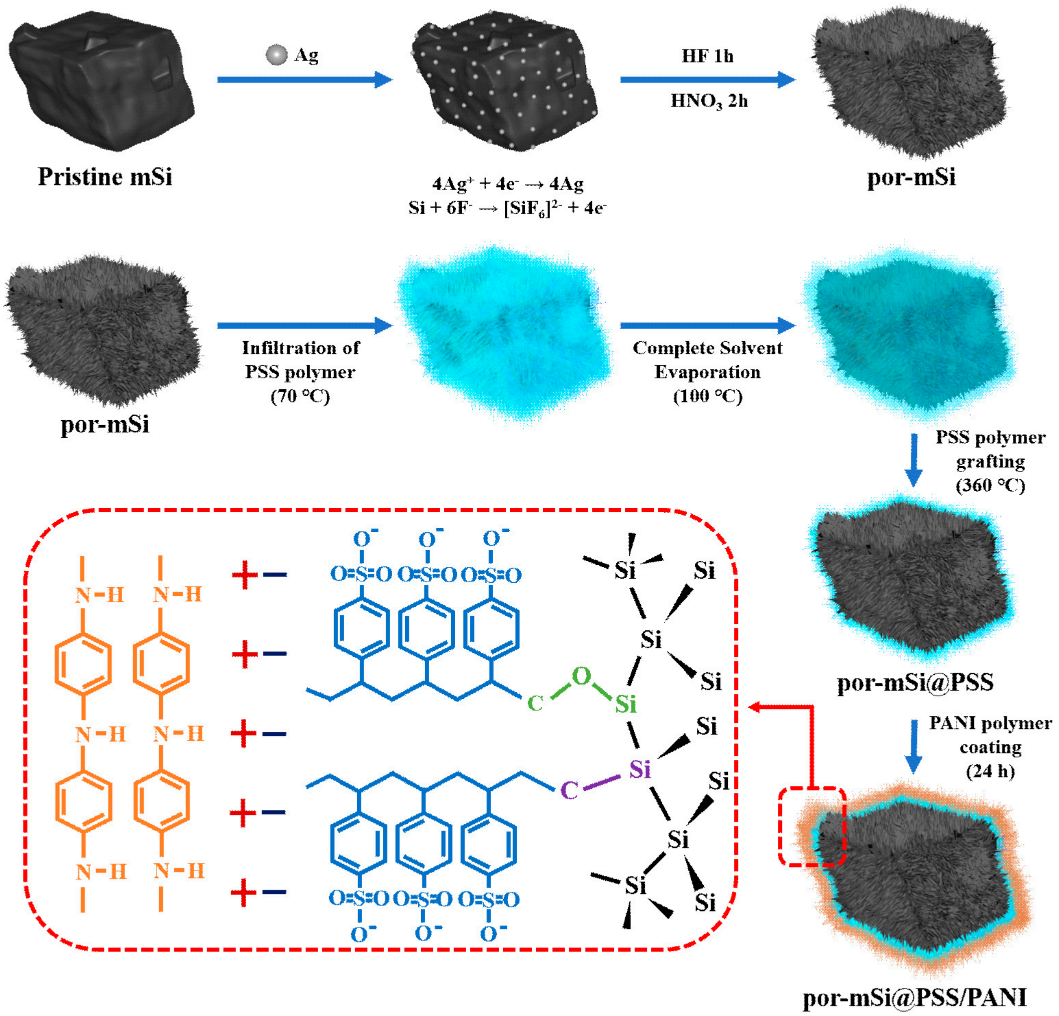

Scheme S1. Schematic of the synthesis of por-mSi, por-mSi@PSS and por-mSi@PSS/PANI.

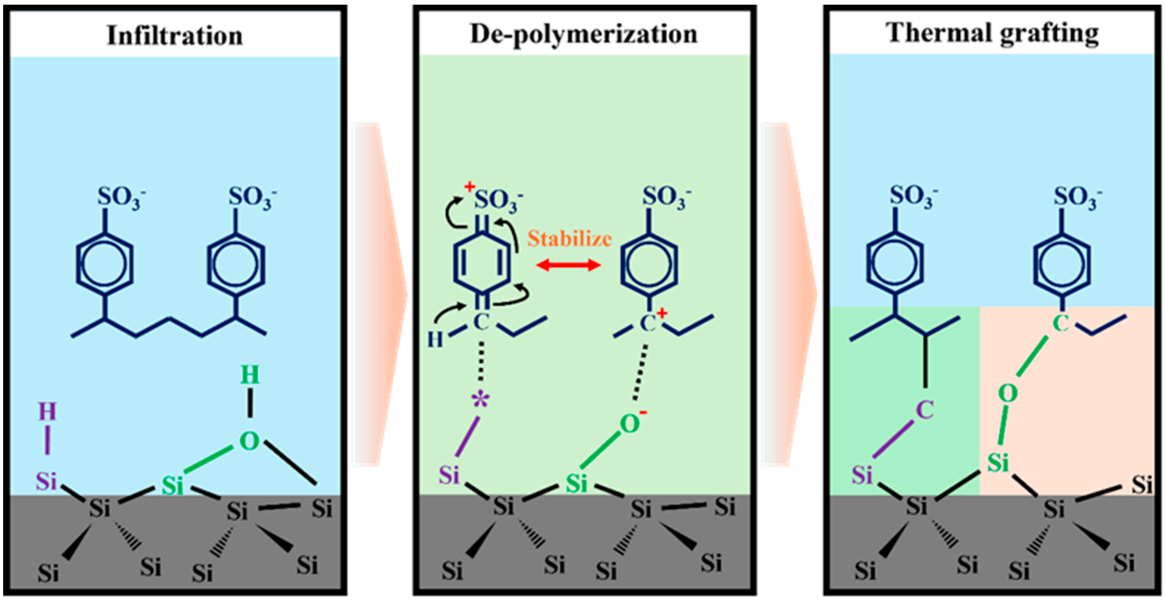

Scheme S2. Proposed thermolytic grafting mechanism for the bonding of styrenic carbon at the interface of porous silicon composites: A: Infiltration, B: De-polymerization, C: Thermal grafting.

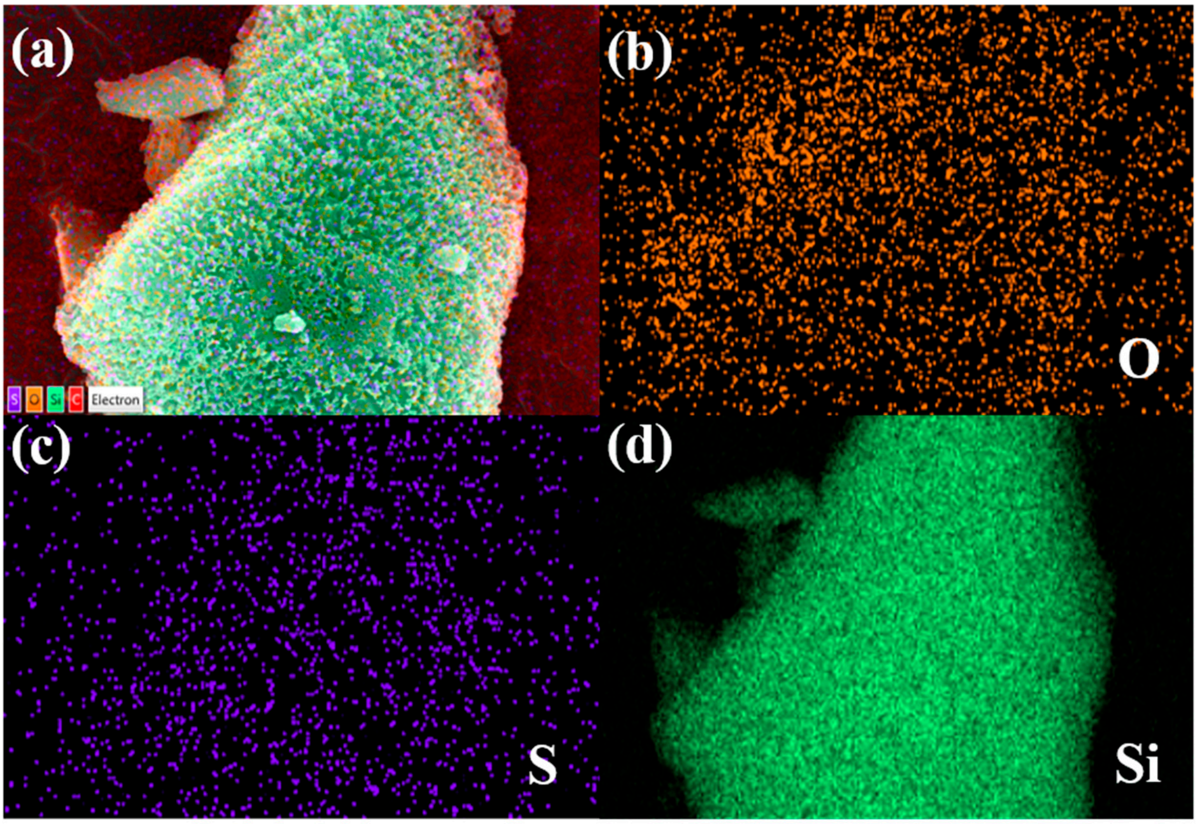

Figure S1. EDS elemental mapping of por-mSi@PSS for (a) full image, (b) O, (c) S and (d) Si elements.

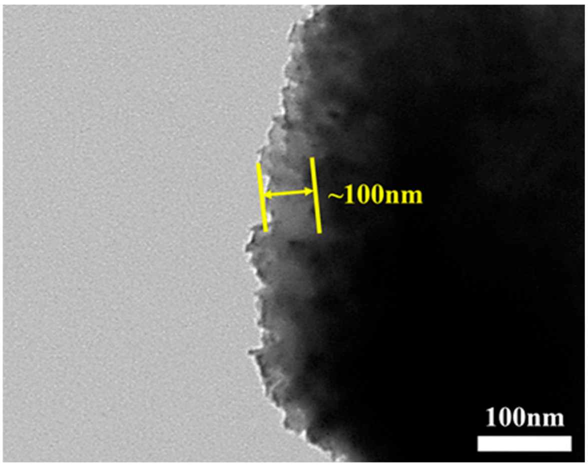

Figure S2. TEM image of por-mSi@PSS/PANI.

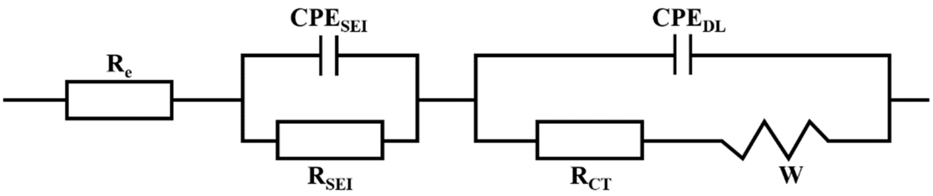

Samples	$R_e(\Omega)$	$R_{SEI+ct}(\Omega)$	$C_1(\mu F)$	$C_2(mF)$	$W(m\Omega\ s^{-1/2})$
mSi	2.211	84.382	2.197	2.755	19.72
por-mSi	1.541	71.184	2.293	2.757	11.63
por-mSi@PSS	2.207	54.769	2.213	2.312	9.699
por-mSi@PSS/PANI	2.671	41.574	2.505	3.245	9.509

Figure S3. Schematic diagram of the equivalent circuit with fitting results for EIS of mSi, por-mSi, por-mSi@PSS and por-mSi@PSS/PANI.

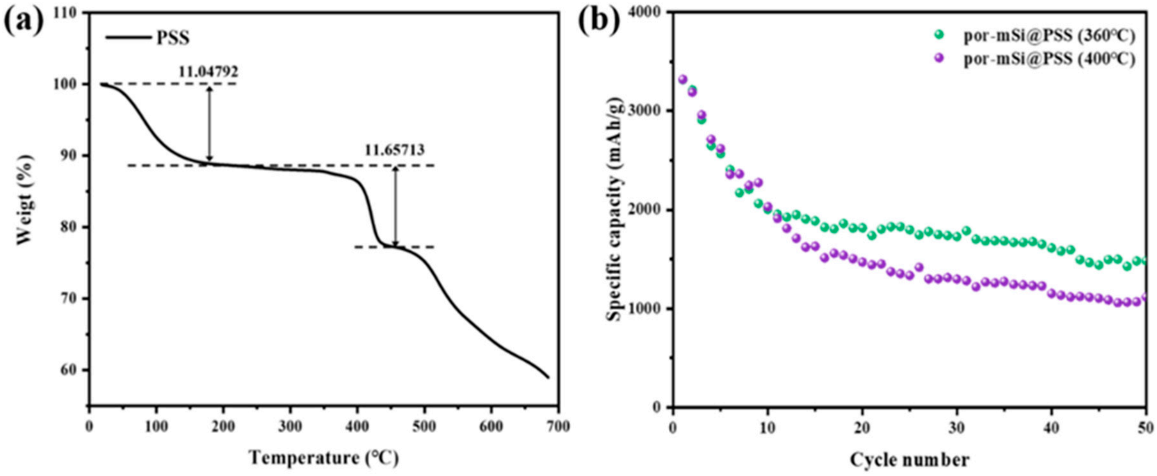

Figure S4. (a) Thermogravimetric Analysis (TGA) of PSS and (b) Cycling performance of por-mSi@PSS (360, 400 ℃) samples at 0.1 A g-1.

Table S1. Summary of literature on the electrochemical performances of carbon-coated Si anode materials

Anode material	Process	Temp (°C)	Si (wt%)	Rate capability[a] (mAh g^{-1}/A g^{-1})	Cyclability[b] (mAh g^{-1}@ cycles)	Ref.
**Si/C@graphite**	Self-assembly	900	6.7	138/1	428@100	[67]
**P2–1050-Si@V@C**	Pyrolysis	850	23.0	743/2	755@200	[68]
**Si-C microsphere**	Poly-condensation	1000	12.3	582/22	558@300	[69]
**Si-CNT**	Chemical dealloying	600	47.3	510/5	1275@100	[70]
**N-Si/C**	Thermal annealing	1000	65.38	877/1	1238@100	[71]
**Pitch-Si-0.5/C**	electro spraying and calcination	800	60.0	406/2	929@200	[72]
**Si@C-G**	Ball milling and pyrolysis	1000	10.0	1117.1/2	80@250	[73]
**Si+rGO@DFAT-C**	Pyrolysis	600	94.7	1126/2	968@200	[74]
**Si/GNs**	Magnesiothermic	650	31.1	675/5	1055.8@100	[75]
**Si@NG**	Pyrolysis	1100	94	388/16	1321@100	[76]
**Si/C microsphere**	CVD	900	16.5	375/1	569@50	[77]
**N-doped graphene @g-C_3N_4/MoS_2**	Hydrothermal	550	-	130/8	855@100	[78]
**por-mSi@PSS/PANI**	**Thermolytic grafting**	**360**	**98.85**	**904/4**	**1398@100 [Figure 6c]**	**This work**

[a] Rate capability was extracted from literature as specific capacity at the highest current density
[b] Cyclability was extracted from literature as final specific capacity versus cycle number

References for Table S1

[67] Wang, Z.; Mao, Z.; Lai, L.; Okubo, M.; Song, Y.; Zhou, Y.; Liu, X.; Huang, W. Sub-micron silicon/pyrolyzed carbon@natural graphite self-assembly composite anode material for lithium-ion batteries. *Chem. Eng. J.* **2017**, *313*, 187-196, https://doi.org/10.1016/j.cej.2016.12.072.

[68] He, Y.; Han, F.; Wang, F.; Tao, J.; Wu, H.; Zhang, F.; Liu, J. Optimal microstructural design of pitch-derived soft carbon shell in yolk-shell silicon/carbon composite for superior lithium storage. *Electrochim. Acta* **2021**, *373*, 137924, https://doi.org/10.1016/j.electacta.2021.137924.

[69] Li, J.-Y.; Li, G.; Zhang, J.; Yin, Y.-X.; Yue, F.-S.; Xu, Q.; Guo, Y.-G. Rational design of robust Si/C microspheres for high-tap-density anode materials. *ACS Appl. Mater. Interfaces* **2019**, *11*, 4057-4064, https://doi.org/10.1021/acsami.8b20213.

[70] Yi, Z.; Lin, N.; Zhao, Y.; Wang, W.; Qian, Y.; Zhu, Y.; Qian, Y. A flexible micro/nanostructured Si microsphere cross-linked by highly-elastic carbon nanotubes toward enhanced lithium ion battery anodes. *Energy Storage Mater.* **2019**, *17*, 93-100, https://doi.org/10.1016/j.ensm.2018.07.025.

[71] Hsieh, C.-C.; Liu, W.-R. Effects of nitrogen doping on Si/carbon composite anode derived from Si wastes as potential active materials for Li ion batteries. *J. Alloys Compd.* **2019**, *790*, 829-836, https://doi.org/10.1016/j.jallcom.2019.03.242.

[72] Chen, C.-Y.; Liang, A.-H.; Huang, C.-L.; Hsu, T.-H.; Li, Y.-Y. The pitch-based silicon-carbon composites fabricated by electrospraying technique as the anode material of lithium ion battery. *J. Alloys Compd.* **2020**, *844*, 156025, https://doi.org/10.1016/j.jallcom.2020.156025.

[73] Zhang, W.; Fang, S.; Wang, N.; Zhang, J.; Shi, B.; Yu, Z.; Yang, J. A compact silicon–carbon composite with an embedded structure for high cycling coulombic efficiency anode materials in lithium-ion batteries. *Inorg. Chem. Front.* **2020**, *7*, 2487-2496, https://doi.org/10.1039/D0QI00302F.

[74] Wang, Q.; Meng, T.; Li, Y.; Yang, J.; Huang, B.; Ou, S.; Meng, C.; Zhang, S.; Tong, Y. Consecutive chemical bonds reconstructing surface structure of silicon anode for high-performance lithium-ion battery. *Energy Storage Mater.* **2021**, *39*, 354-364, https://doi.org/10.1016/j.ensm.2021.04.043.

[75] Zhang, Y.; Wang, Z.; Hu, K.; Ren, J.; Yu, N.; Liu, X.; Wu, G.; Liu, N. Anchoring silicon on the basal plane of graphite via a three-phase heterostructure for highly reversible lithium storage. *Energy Storage Mater.* **2021**, *34*, 311-319, https://doi.org/10.1016/j.ensm.2020.10.002.

[76] Zhou, Y.; Yang, Y.; Hou, G.; Yi, D.; Zhou, B.; Chen, S.; Lam, T.D.; Yuan, F.; Golberg, D.; Wang, X. Stress-relieving defects enable ultra-stable silicon anode for Li-ion storage. *Nano Energy* **2020**, *70*, 104568, https://doi.org/10.1016/j.nanoen.2020.104568.

[77] Ren, W.; Zhang, Z.; Wang, Y.; Tan, Q.; Zhong, Z.; Su, F. Preparation of porous silicon/carbon microspheres as high performance anode materials for lithium ion batteries. *J. Mater. Chem. A* **2015**, *3*, 5859-5865, https://doi.org/10.1039/C4TA07093C.

[78] Hou, Y.; Li, J.; Wen, Z.; Cui, S.; Yuan, C.; Chen, J. N-doped graphene/porous g-C3N4 nanosheets supported layered-MoS2 hybrid as robust anode materials for lithium-ion batteries. *Nano Energy* **2014**, *8*, 157-164, https://doi.org/10.1016/j.nanoen.2014.06.003.
